# The Utility of Deep Learning in Breast Ultrasonic Imaging: A Review

**DOI:** 10.3390/diagnostics10121055

**Published:** 2020-12-06

**Authors:** Tomoyuki Fujioka, Mio Mori, Kazunori Kubota, Jun Oyama, Emi Yamaga, Yuka Yashima, Leona Katsuta, Kyoko Nomura, Miyako Nara, Goshi Oda, Tsuyoshi Nakagawa, Yoshio Kitazume, Ukihide Tateishi

**Affiliations:** 1 Department of Diagnostic Radiology, Tokyo Medical and Dental University, Tokyo 113-8510, Japan; fjokmrad@tmd.ac.jp (T.F.); kubotard@dokkyomed.ac.jp (K.K.); ooymmrad@tmd.ac.jp (J.O.); ymgdrnm@tmd.ac.jp (E.Y.); 11.ruby.89@gmail.com (Y.Y.); leonah@jcom.home.ne.jp (L.K.); nomura.kyoko@kameda.jp (K.N.); miyako641@gmail.com (M.N.); ktzmmrad@tmd.ac.jp (Y.K.); ttisdrnm@tmd.ac.jp (U.T.); 2 Department of Radiology, Dokkyo Medical University, Tochigi 321-0293, Japan; 3 Department of Breast Surgery, Tokyo Metropolitan Cancer and Infectious Diseases Center Komagome Hospital, Tokyo 113-8677, Japan; 4 Department of Surgery, Breast Surgery, Tokyo Medical and Dental University, Tokyo 113-8510, Japan; oda.srg2@tmd.ac.jp (G.O.); nakagawa.srg2@tmd.ac.jp (T.N.)

**Keywords:** breast, ultrasound, deep learning, machine learning, artificial intelligence, neural network

## Abstract

Breast cancer is the most frequently diagnosed cancer in women; it poses a serious threat to women’s health. Thus, early detection and proper treatment can improve patient prognosis. Breast ultrasound is one of the most commonly used modalities for diagnosing and detecting breast cancer in clinical practice. Deep learning technology has made significant progress in data extraction and analysis for medical images in recent years. Therefore, the use of deep learning for breast ultrasonic imaging in clinical practice is extremely important, as it saves time, reduces radiologist fatigue, and compensates for a lack of experience and skills in some cases. This review article discusses the basic technical knowledge and algorithms of deep learning for breast ultrasound and the application of deep learning technology in image classification, object detection, segmentation, and image synthesis. Finally, we discuss the current issues and future perspectives of deep learning technology in breast ultrasound.

## 1. Introduction

Breast cancer is the most common cancer and the second leading cause of cancer-related death in women [1]. Owing to its advantages, such as safety, convenience, and low cost, ultrasound is used to detect and diagnose breast lesions when abnormalities are identified by other imaging modalities or on palpation [2]. In addition, breast ultrasound is expected to emerge as a complementary screening method for women with mammographically dense breasts, and the screening practice is expected to detect tumors at an early stage and reduce breast cancer mortality in women [3].

The use of breast ultrasound is increasing, and radiologists and clinicians spend significant time examining huge volumes of breast ultrasonic images. This has become a major problem in many countries because it leads to increased medical costs and worsens the patient case. Although breast ultrasonic imaging is performed using the Breast Imaging Reporting and Data System [4], it is difficult to maintain control over the accuracy of diagnostic imaging in areas in which experts are scarce.

In recent years, artificial intelligence (AI), especially deep learning methods, has accomplished outstanding performance in automatic speech recognition, image recognition, and natural language processing. The applications of deep learning in biomedical fields include all the medical levels, from genomic applications such as gene expression to public medical health management such as prediction of demographic information or infectious disease epidemics [5,6]. During the previous few decades, advances in high-throughput technology have significantly increased the amount of biomedical data. These biomedical data require effective and efficient computational tools for their storage, analyses, and interpretation. Deep learning-based algorithmic frameworks can resolve these challenges [7,8].

Furthermore, research on deep learning technology has been actively increasing in the field of medical imaging. It has been applied to radiologic images, for instance, to detect tuberculosis on chest radiographs, detect and diagnose lung nodules on chest CT, and segment brain tumor on MRI [9,10,11]. Furthermore, deep learning has proven useful in the field of breast imaging, and deep learning-based diagnostic support systems are becoming more commonly available [12,13,14,15,16,17]. The technology is also used in pathological imaging and is reportedly useful in counting the number of cells or mitosis, grading tumor tissue, segmenting the nucleus, and estimating the risk of recurrence with Oncotype DX [18,19,20,21].

Deep learning has also been used in breast ultrasonic imaging and will have a great influence in the future. Effective use of deep learning is expected to assist clinicians and help improve the standard of care. Health care workers involved in breast care must understand the current state of deep learning in breast ultrasound and consider how it will evolve.

This review discusses the basic technical knowledge and algorithms of deep learning for breast ultrasound and its application in image classification, segmentation, and image synthesis. Finally, we discuss current issues and future perspectives of deep learning in breast ultrasound.

## 2. What Are AI, Machine Learning, and Deep Learning?

**Figure 1:**
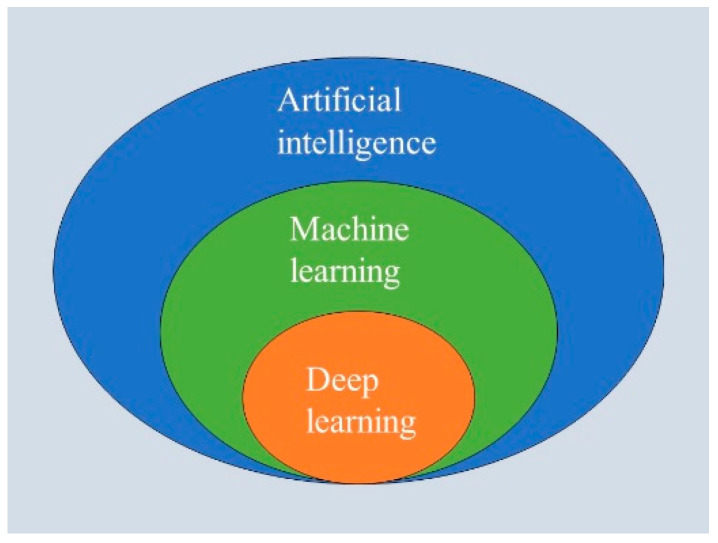
Schematic diagram of artificial intelligence, machine learning, and deep learning.

AI is a broad area of computer science related to building smart machines that can perform tasks that normally require human intelligence [22]. Machine learning is a term introduced by Arthur Samuel in 1959 to describe one of the technologies of AI (Figure 1). Machine learning provides a system with the ability to automatically learn and improve from experience without explicit programming. In classic machine learning, expert humans discern and encode features that appear distinctive in the data, and statistical techniques are used to organize or segregate the data based on these features [22,23].

Deep learning is part of a broader family of machine learning methods based on artificial neural networks with representation learning (Figure 1). It can make flexible decisions according to the situation by learning a large amount of data and automatically extracting common feature quantities [23,24]. Deep learning can generally be viewed as applying single-layer traditional machine learning methods (i.e., projection, convolution, hyperplane separation, and dot product) in a sequential, layer-wise manner, thus resulting in computational architectures with a depth dimension. Fully automatic training via the back-propagation algorithm is based on gradient descent, where graphics processing units are generally necessary to manage computational complexity [24].

**Figure 2:**
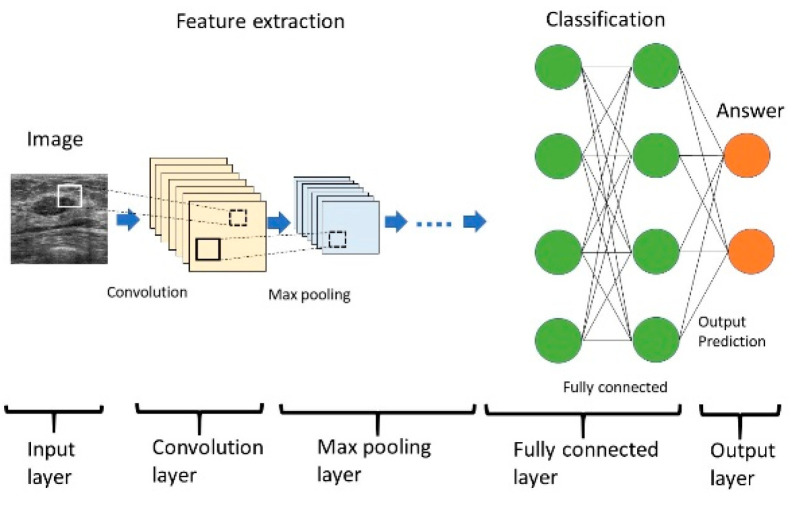
Structure of a convolutional neural network.

Deep learning has dramatically improved research in areas such as speech recognition, visual image recognition, and object detection [24]. In image processing, the deep learning architecture called a convolutional neural network (CNN) has become its mainstream feature. Several types of CNNs have been developed. CNN consists of input and output layers, along with the important components of the convolution, max pooling, and fully connected layers (Figure 2) [25]. CNN typically includes multiple convolutional and pooling layers that facilitate the learning of more and more abstract features. The convolution layer extracts a feature from the input image and passes these results to the next layer. Convolution uses small squares of the input data to learn the features of the image, thus maintaining the relationships between the pixels and resulting in activation. Repeated application of the same filter to the input produces a map of activation, a feature map, which reveals the location and intensity of the features detected in the input, such as the image [25,26]. The pooling layers section reduces the spatial size of the activation maps for minimizing the likelihood of overfitting. Spatial pooling is called subsampling or downsampling, which reduces the dimensionality of each map but retains important information. Although there are several types of spatial pooling, max pooling has been the most common type [25,26]. The purpose of a fully connected layer is to obtain the results of the convolutional/pooling process and use them to classify the images into labels. The output of the convolution/pooling is flattened into a single vector of values. Fully connected layers connect all neurons in one layer to all neurons in another layer. The inputs from the neurons in the previous layer to those in the next layer are combined into a signal by the connection, a linear transformation process, and the signal is then output to the next layer of neurons via an activation function, a non-linear transformation. The rectified linear unit (Relu) function is commonly used as the activation function [25,26]. The output layer is the last layer that produces the given outputs for the program. The neurons are designed to rationalize and improve the end result of the iterative process, and the softmax function is typically used for multiple classification tasks [22,25].

With increasing depth and complexity, CNN offers amazing performance evolution compared with traditional computer vision technology [25]. CNN has attracted significant attention since winning the international image classification competition ImageNet Large Scale Visual Recognition Challenge (ILSVRC) in 2012 [22,23].

AI is a broad area of computer science. Machine learning provides a system with the ability to automatically learn and improve from experience without explicit programming. Deep learning is part of a broader family of machine learning methods based on artificial neural networks.

CNN consists of multiple layers. Input data are first processed with convolutional and max pooling layers for feature extraction. These data are further processed in fully connected layers for classification, and the final prediction is output. Layers between the input and output layers are called hidden layers.

Learning methods include supervised learning, which learns a large amount of data and automatically acquires its characteristics; unsupervised learning, which classifies data in various dimensions; and reinforcement learning, which obtains the correct answer via a repeated trial-and-error method.

We must collect datasets (training, validation, and test data) to perform deep learning. The model first fits the training dataset. This is a set of examples used to match model parameters (such as the weight of connections between neurons in an artificial neural network). The fitted model is then used to predict the response of the observations of the second dataset called the validation dataset. Finally, the test dataset is used to provide an unbiased evaluation of the final model that fits the training dataset [22].

Several techniques are available for efficient deep learning. Data augmentation is one solution when insufficient enough training data are available for constructing a deep learning model. Image datasets can be augmented via flipping, cropping, rotating, and adjusting the contrast, sharpness, and white balance. The diversification of training datasets helps prevent overfitting and enhances model generalization [25]. Transfer learning is an efficient learning method that makes one trained model useful for another area. Transfer learning has the advantages of being able to learn quickly and achieving high accuracy even with small amounts of data [25].

## 3. Development of AI Research on Breast Ultrasound

The development of AI research on breast ultrasound has led to an increase in publications in this area. PubMed was searched through 31 October 2019 to extract articles on imaging and AI used for breast ultrasound using the following search query: “artificial intelligence” or “machine learning” OR “deep learning” AND “breast ultrasound.” Among these articles, only those that described studies on AI for breast imaging were selected.

**Figure 3:**
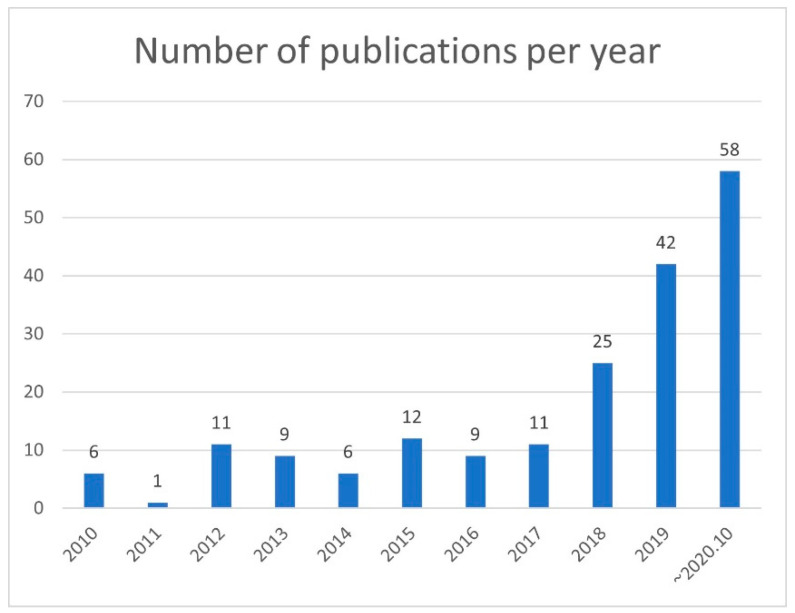
Number of publications per year.

The annual number of publications did not change from 2010 to 2017, but it increased significantly from 2018 to 2020, with more than 20 publications in 2018 and more than 40 publications in 2019. Meanwhile, 50 papers were published in the first 10 months of 2020 (Figure 3). Many of these studies described imaging classification, object detection, segmentation, and synthetic imaging of breast lesions.

The number of publications indexed on PubMed was obtained using the following search query: “artificial intelligence” or “machine learning” OR “deep learning” AND “breast ultrasound.” PubMed was accessed on 31 October 2020. Since 2018, the number of papers on AI of breast ultrasound has increased rapidly.

## 4. Image classification

**Figure 4:**
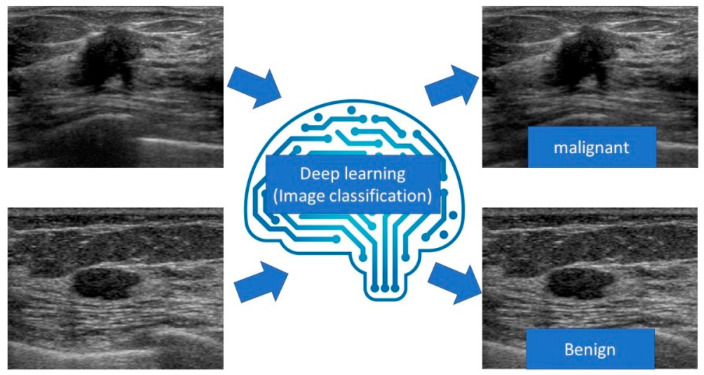
Diagram of image classification for breast ultrasound.

Image classification is a method for identifying and predicting what an image represents (Figure 4). The development of machine learning, especially deep learning with CNN, has facilitated the creation of highly accurate models. Since AlexNet [27] won the ILSVRC with overwhelming results, deep learning techniques have acquired a leading role in image classification. Subsequently, capable networks such as VGGNet [28], GoogLeNet [29], ResNet [30], and DenseNet [31] have been developed successively with deeper layers of CNN.

In the image classification of breast ultrasound, many reports discussed the distinction between benign and malignant lesions on B-mode images (Table 1). Han et al. collected a large number of B-mode images to train a deep neural network with 3154 malignant and 4254 benign samples using GoogLeNet to distinguish the malignancy of breast masses on ultrasound. They reported that the deep learning model had an accuracy of 91%, a sensitivity of 86%, a specificity of 93%, and an area under the curve (AUC) of >0.9 [32].

We also collected B-mode images of 480 benign and 467 malignant masses as training data. A deep learning model was constructed using the CNN architecture GoogLeNet Inception v2, and 48 images of benign masses and 72 images of malignant masses were analyzed as the test data. The deep learning model had an accuracy of 95.8%, a sensitivity of 87.5%, a specificity of 92.5%, and an AUC of 0.913, and its diagnostic performance was equal or superior to that of radiologists [33].

Mango et al. evaluated the utility of Koios DS Breast, a machine learning-based diagnostic support system (Koios, https://koiosmedical.com/), by performing reading tests using the ultrasound images of 900 breast lesions. Among 15 physicians, the mean reader AUC for cases reviewed using ultrasound only was 0.83 versus 0.87 for ultrasound plus Koios DS Breast. Thus, Koios DS Breast improved the accuracy of sonographic breast lesion assessment while reducing inter- and intra-observer variability [34]. This diagnostic support system has been approved by the US Food and Drug Administration (FDA) and commercially launched.

Zhang et al. built a deep learning architecture and evaluated its performance in the differentiation of benign and malignant breast tumors on a set of shear wave elastography (SWE) images using 135 benign and 92 malignant tumors. The deep learning architecture displayed good classification performance, with an accuracy of 93.4%, a sensitivity of 88.6%, a sensitivity of 97.1%, and an AUC of 0.947 [35].

We also gathered 158 images of benign masses and 146 images of malignant masses as training data for SWE. Deep learning models were constructed using several state-of-the-art architectures. We analyzed the SWE images of 38 benign and 35 malignant masses as test data. The best model (DenseNet 169) had a sensitivity of 85.7%, a specificity of 78.9%, and an AUC of 0.898. The deep learning models had equal or better diagnostic performance than radiologist readings [36].

Coronado-Gutiérrez et al. developed quantitative ultrasound image analysis techniques using deep learning to noninvasively diagnose axillary lymph node involvement in breast cancer using 118 lymph node ultrasound images. The achieved accuracy of this method was 86.4%, and its sensitivity and specificity were 84.9 and 87.7%, respectively [37].

After inputting an image, the deep learning algorithm identifies and predicts what the image represents.

## 5. Object Detection

**Figure 5:**
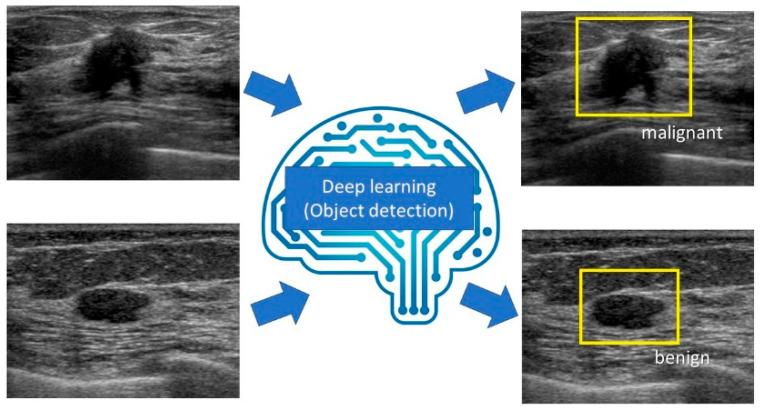
The diagram of object detection for breast ultrasound.

Object detection refers to detecting the location and category (class) of a defined object in an image (Figure 5). Similar to image classification, with the development of deep learning with CNN, faster and more accurate models have been created. In 2014, Regions with Convolutional Neural Networks (R-CNN) was developed to successfully apply the CNN algorithm to the task of object detection [38]. Subsequently, high-speed, high-precision object detection models such as Spatial Pyramid Pooling (SPP)-net [39], Fast R-CNN [40], Faster R-CNN [41], You Only Look Once (YOLO) [42], Single Shot MultiBox Detector (SSD) [43], feature pyramid networks [44], and RetinaNet [45] were developed.

Cao et al. studied the existing state-of-the-art CNN methods (Fast R-CNN, Faster R-CNN, YOLO, and SSD) for breast lesion detection using breast ultrasound images. They collected a dataset consisting of 579 benign and 464 malignant lesions and submitted the ultrasound images to manual annotation by experienced clinicians. They found that YOLO and SSD perform significantly better than the other methods in detecting breast lesions [46].

Jiang et al. compared the diagnostic accuracy and interpretation time of screening automated breast ultrasound (ABUS) for women with dense breast tissue with and without the use of the deep learning-based computer-aided detection (CAD) system QVCAD (QViewMedical, https://www.qviewmedical.com/); 18 radiologists interpreted a test set of 185 screening ABUS images (52 with and 133 without breast cancer). The AUC was 0.848 with the CAD system, compared with 0.828 without the CAD system, which was statistically noninferior. The mean interpretation time was 3.55 min per case without the CAD system, compared with 2.4 min with the CAD system. This diagnostic support system has been approved by the FDA and reported to be useful in the US [47]. Yang et al. [48] and Xu et al. [49] also demonstrated that CAD systems improve the diagnostic performance for ABUS interpretation (Table 2).

After inputting an image, deep learning systems detect the location and category (class) of a defined object in the image.

## 6. Segmentation

**Figure 6:**
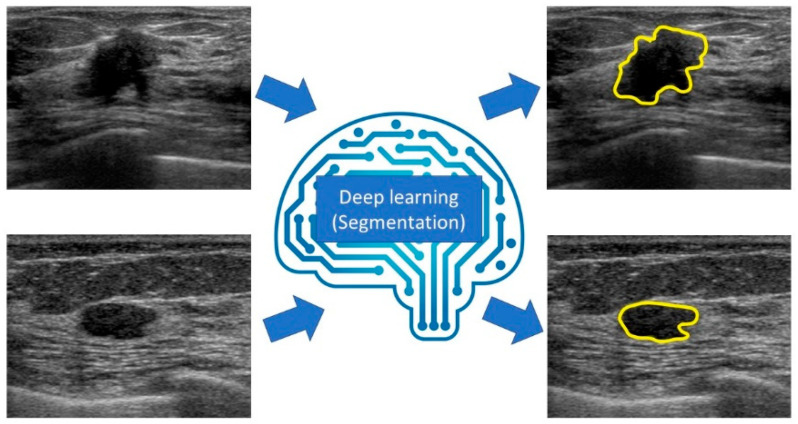
Diagram of segmentation for breast ultrasound.

Semantic segmentation is a method that can associate labels and categories with all pixels in an image and divide the object into multiple regions at the pixel level (Figure 6).

Models based on the CNN architecture that enable high-precision and high-speed semantic segmentation, such as Fully Convolutional Network (FCN) [50], Segnet [51], and U-net [52], have been developed. They are widely used in various industries that require high-precision image mapping, such as medical imaging, autonomous driving, industrial inspection, and satellite imagery. Zhang et al. developed Residual-Dilated-Attention-Gate-U-net, an improved version of U-net, and performed segmentation of lesions on breast ultrasound images. Their model displayed better breast lesion segmentation (precision rate: 88.58%, recall rate: 83.19%, F1 score: 84.78) than the traditional CNN model [53].

Hu et al. proposed combining a dilated fully convolutional network with a phase-based active contour model for automatic tumor segmentation. Their models exhibited high robustness, accuracy, and efficiency (Dice similarity coefficient: 88.97%, Hausdorff distance: 35.54 pixels, mean absolute deviation: 7.67 pixels), similar to the manual segmentation results [54].

Kumar et al. developed a Multi U-net algorithm based on CNNs that automatically segments breast masses. The model effectively segmented the breast masses, achieving a mean Dice coefficient of 0.82, a true positive fraction of 0.84, and a false positive fraction of 0.01 [55] (Table 3).

After inputting an image, the deep learning system associates labels and categories with every pixel in the image and divides the object into multiple areas at the pixel level.

## 7. Image synthesis

**Figure 7:**
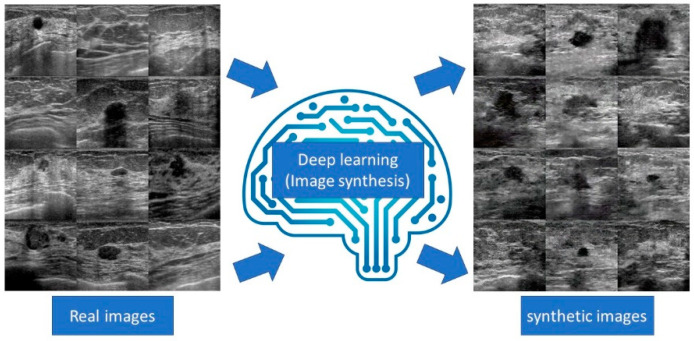
Diagram of image synthesis for breast ultrasound.

Image synthesis involves the generation of realistic images using a computer algorithm. This method is useful for improving image quality by reconstructing and generating training data by generating virtual images (Figure 7). One of the most interesting breakthroughs in the field was the advent of generative adversarial networks (GANs), which consist of effective machine learning frameworks used to train unsupervised generative models [56]. GAN is a special type of neural network model in which two networks are trained simultaneously, with one focusing on image generation and the other focusing on discrimination. Variational autoEncoder (VAE) is another effective generative model. Autoencoder (AE) is a type of neural network that learns the data encodings from the dataset in an unsupervised manner. Basically, VAE contains two parts; one is an encoder that learns efficient data encoding from the dataset and passes it into a bottleneck architecture and the other is a decoder that uses latent space in the bottleneck layer to regenerate images similar to that in the dataset. VAE differs from AE in that it provides a statistical manner to describe the samples of the dataset in latent space. Therefore, in VAE, the encoder outputs the probability distribution to the bottleneck layer instead of a single output value [57].

In recent years, a competent generative model based on deep learning was developed, and its effectiveness was confirmed. Image reconstruction using its technology makes it possible to generate images with few artifacts. In addition, a high-frame-rate image can be generated by performing image interpolation. Because the virtual image generated by the generative model does not contain personal information, it is considered useful for research and education that is open to the public [58]. We succeeded in generating realistic virtual images of breast tumors and virtual interpolation images of tumor development using deep convolutional GANs [59,60]. In addition, we used a GAN-based anomaly detection model to distinguish normal tissues from benign and malignant masses. The model had a sensitivity of 89.2%, a specificity of 90.2%, and an AUC of 0.936 [61]. Han et al. proposed a semi-supervised segmentation network based on GANs. They found that the model achieved higher segmentation accuracy than state-of-the-art semi-supervised segmentation methods [62].

After inputting a real image, the deep learning machine synthesizes realistic virtual images.

## 8. Discussion

In this study, we explained the basic technical knowledge and algorithms of deep learning in breast ultrasound and discussed current research trends. Deep learning techniques in breast ultrasound have evolved in recent years, and the number of related publications has increased. Research on deep learning models for breast ultrasound is actively being conducted, but a few computer-aided diagnosis support systems based on deep learning have been approved by public institutions. To our knowledge, three machine learning- or deep learning-based diagnostic imaging support systems for mammograms have been approved by the FDA: HealthMammo (Zebra Medical Vision, https://www.zebra-med.com/), ProFoundAI (iCAD, https://screenpoint-medical.com/), and Transpara (ScreenPoint Medical, https://screenpoint-medical.com/) [63]. These systems detect lesions such as masses and calcifications and score the malignant probability of the lesion. In breast ultrasound, as previously mentioned, two breast ultrasound systems, namely Smart Ultrasound (Koios) for 2D-B-mode imaging and QVCAD (QViewMedical) for ABUS, have been authorized by the FDA.

Contrarily, in Japan, no deep learning-based diagnostic imaging support systems for breast imaging have gained approval from the Pharmaceuticals and Medical Devices Agency. However, we believe that it will be possible to perform breast imaging in Japan using deep learning-based diagnostic imaging support systems by importing foreign-developed systems or developing a system unique to the country in the near future.

However, there are some obstacles to the introduction of deep learning-based diagnostic support systems for breast imaging. First, it is necessary to clarify who is responsible for the diagnosis. Previous studies revealed that even efficient deep learning models can misdiagnose lesions similarly to humans. At present, deep learning systems remain a supplementary modality for doctors, but there will be debates concerning whether these systems can replace doctors if their accuracy is improved in the future. If a deep learning system makes a serious mistake, it will be necessary to conclude whether the developer, seller, or doctor is responsible for the misdiagnosis.

Diagnostic systems based on deep learning have a complex structure. Even if the results are correct, doctors do not know the basis on which the deep learning system reached the diagnosis. This is called the black box problem. It will be necessary to develop and research deep learning systems that can both provide a diagnosis and clarify the reason for the same [64,65].

The diagnostic performance of deep learning models can be significantly reduced if there is a significant difference between the deep learning system-trained and deep learning system-adapted populations. To use deep learning diagnostics properly, it will be necessary to understand in what population (e.g., prevalence, severity, and type of disease) and for what purpose (e.g., screening and scrutiny) the deep learning system was trained.

It is expected that deep learning support for image classification, object detection, segmentation, and image synthesis will improve diagnostic performance, streamline physician work, and reduce health care costs in the screening, differential diagnosis, efficacy assessment, and surveillance of breast cancer. Physicians will be able to spend more time communicating with patients and doctors in other departments, which will improve the quality of hospital care. Although some competent deep learning systems have been developed, the systems are only applicable for a limited purpose or modality. In the future, it will be necessary to develop a highly versatile deep learning system that combines multiple datasets such as images from several modalities, blood test data, and clinical information such as patients’ symptoms to comprehensively diagnose diseases and manage treatment. A high-performance system may be better than clinicians and even replace them. Clinicians with a better understanding of deep learning and willingness to use such systems will be able to play a leading role in this imminent change.

## 9. Conclusions

Deep learning represents a powerful, efficient, highly accurate tool that reduces physicians’ workload. Deep learning of breast ultrasound images is expected to be further developed and clinically applied in various situations such as image classification, object detection, and segmentation. We must have basic knowledge of deep learning, anticipate the problems that will occur when it is introduced, and prepare to address those problems.

## Figures and Tables

**Table 1:** Deep learning models for image classification.

Purpose (Type of Image)	Model	Number of Training Set Images	Number of Test Set Images	Result	Study
Breast lesions (B-mode images)	GoogLeNet	6579	829	Sensitivity: 86% Specificity: 96% Accuracy: 90% AUC: 0.9	[32]
Breast lesions (B-mode image)	GoogLeNet Inception v2	937	120	Sensitivity: 95.8% Specificity: 87.5% Accuracy: 92.5% AUC: 0.913	[33]
Breast lesions via CAD (B-mode image)	Koios DS	Over 400,000	900	AUC without CAD: 0.83 AUC with CAD: 0.87	[34]
Breast lesions (SWE image)	PGBM and RBM	227	Five-fold cross-validation	Sensitivity: 88.6% Specificity: 97.1% Accuracy: 93.4% AUC: 0.947	[35]
Breast lesions (SWE image)	DenseNet 169	304	73	Sensitivity: 85.7% Specificity: 78.9% AUC: 0.898	[36]
Axillary lymph nodes (B-mode image)	VGG-M model	118	Five-fold cross-validation	Sensitivity: 84.9% Specificity: 87.7% Accuracy: 86.4% AUC: 0.937	[37]

CAD, computer-assisted diagnosis; PGBM, point-wise gated Boltzmann machine; RBM, restricted Boltzmann machine; AUC, area under the curve.

**Table 2:** Deep learning models for object detection.

Purpose (Type of Image)	Model	Number of Training Set Images	Number of Test Set Images	Result	Study
Object detection of breast lesions (B-mode image)	SSD300	860	183	Precision rate: 96.89% Recall rate: 67.23% F1 score: 79.38%	[46]
Object detection of breast lesions by CAD (ABUS image)	QVCAD	Over 20,000	185	AUC without CAD: 0.828 AUC with CAD: 0.848	[47]
Object detection of breast lesions by CAD (ABUS image)	QVCAD	Over 20,000	1485	AUC without CAD: 0.88 AUC with CAD: 0.91 Sensitivity without CAD: 67% Sensitivity with CAD: 88%	[48]
Object detection of breast lesions by CAD (ABUS image)	QVCAD	Over 20,000	1000	AUC without CAD: 0.747 AUC with CAD: 0.784	[49]

CAD, computer-assisted diagnosis; AUC, area under the curve; ABUS, automated breast ultrasound.

**Table 3:** Deep learning models for segmentation.

Purpose (Type of image)	Model	Number of Training Set Images	Number of Test Set Images	Result	Study
Segmentation of breast lesions (B-mode image)	RDAU-NET	857	205	Precision rate: 88.58% Recall rate: 83.19% F1 score: 84.78	[53]
Segmentation of breast lesions (B-mode image)	Combining DFCN with a PBAC model	400	170	Dice similarity coefficient: 88.97% Hausdorff distance: 35.54 pixels Mean absolute deviation: 7.67 pixels	[54]
Segmentation of breast lesions (B-mode image)	Multi U-net algorithm	372	61	Mean Dice coefficient: 0.82 True positive fraction: 0.84 False positive fraction: 0.01	[55]

CAD, computer-assisted diagnosis; RDAU-NET, Residual-Dilated-Attention-Gate-U-net; DFCN, dilated fully convolutional network; PBAC, phase-based active contour; AUC, area under the curve.
